# When folding CARDs wins the game: molecular snapshots of lipopolysaccharide sensing by the non-canonical inflammasome

**DOI:** 10.1042/BST20260715

**Published:** 2026-09-10

**Authors:** Jakub Began, Petr Broz

**Affiliations:** Department of Immunobiology, University of Lausanne, Switzerland

**Keywords:** caspase-4, host-pathogen interactions, inflammasome, innate immunity, lipopolysaccharides, membrane

## Abstract

Inflammatory caspases play essential roles in regulating IL-1 cytokine processing and secretion, as well as in inducing pyroptosis as part of the innate immune response to pathogens and other noxious stimuli. While the activation of caspase-1 is controlled by dedicated pattern recognition receptors, human caspase-4 and caspase-5, and murine caspase-11 function themselves as direct sensors for lipopolysaccharide (LPS), the immunostimulatory component of the Gram-negative bacterial outer membrane. These caspases bind LPS through their N-terminal caspase recruitment domain (CARD), which triggers their oligomerization and activation. Recent studies have provided new insights into the molecular basis of LPS recognition by the caspase-4/11 CARD, including its interactions with LPS and other lipid ligands, the evolutionary origins of inflammatory caspases, the structural organization of LPS-caspase complexes, and the regulatory mechanisms that govern CARD-ligand association. In this review, we summarize these advances and discuss their implications for inflammatory caspase activation and innate immune signaling.

## Introduction

The outer bacterial membrane component lipopolysaccharide (LPS) is the principal trigger of the innate immune responses during many Gram-negative bacterial infections [1,2]. The immunostimulatory properties of LPS were first recognized by Richard Pfeiffer over a century ago, and efforts to understand how mammalian cells detect LPS were fundamental in laying the foundations for modern innate immunity and establishing the concept of immune sensors, known as pattern recognition receptors (PRRs), that detect microbe-derived ligands, so-called pathogen-associated molecular patterns (PAMPs) [3,4]. Structurally, LPS consists of three moieties: lipid A, core oligosaccharides, and O-antigen. Of these, lipid A, a β-1′-6-linked glucosamine disaccharide that is commonly hexa-acylated in most bacterial species, constitutes the most conserved and immunostimulatory part of the molecule, whereas core oligosaccharides and O-antigen are highly variable [5].

Human cells express PRRs that can detect LPS either in the extracellular compartment or within the cytosol [6]. Extracellular LPS is detected by Toll-like receptor 4 (TLR4), which engages conserved signaling pathways via the adaptors MyD88 and TRIF to drive the expression of pro-inflammatory cytokines and interferons (IFN) [7,8]. Intracellular LPS, on the other hand, is detected by the non-canonical inflammasome pathway that controls the activation of caspase-4 in humans and caspase-11 in mice [9–12]. Direct binding of LPS to the N-terminal caspase recruitment domain (CARD) of these proteases promotes their autoproteolytic processing, which is needed for activation [13,14]. Once activated, the caspases process IL-1 family cytokines and the pore-forming cell death executioner gasdermin-D (GSDMD), thereby inducing a lytic proinflammatory cell death known as pyroptosis [13,15–18]. These responses serve to alert the immune system and restrict further intracellular replication of pathogens [19].

Recent mechanistic work suggests that these two detection pathways rely on fundamentally different modes of LPS recognition. The TLR4/MD-2 complex contains a hydrophobic pocket that accommodates a single LPS molecule, promoting dimerization of TLR4/MD-2 complexes and initiating downstream signaling [7,20]. In contrast, caspase-4 (-11) has been shown to bind LPS micelles *in vitro* [12,21,22] and is recruited to the surface of cytosolic bacteria in cells [23–25], indicating that it interacts directly with LPS-containing membranes rather than monomeric LPS [22,26]. This mode of detection may represent an evolutionarily more ancient strategy, as Factor C, the LPS-responsive protease of the Limulus Amebocyte Lysate assay, has also been proposed to sense LPS in aggregated form [27,28].

In this review, we discuss recent mechanistic insights into the detection of LPS by the non-canonical inflammasome, with a particular focus on the structural determinants within N-terminal CARD domain of caspase-4/11 that are required for LPS binding and the assembly of LPS-caspase-4/11 complexes. We also review reports of additional lipidic ligands that can modulate non-canonical inflammasome activity, acting either as activators or inhibitors of the pathway.

## The structure of the LPS/caspase-4(-11) complex

The non-canonical inflammasome was discovered in the early 2010s as an intracellular LPS-sensing pathway, essential for anti-microbial defense. Unlike canonical inflammasomes, in which PRRs assemble signaling complexes to activate caspase-1, mouse caspase-11 and human caspase-4/-5 function both as LPS receptors and signaling executioners of the non-canonical inflammasome [9–11,29]. Yet, although the direct interaction of their CARD with LPS was demonstrated over 10 years ago [12], structural insights into the resulting signaling complexes remained scarce until recently.

**Figure 1:**
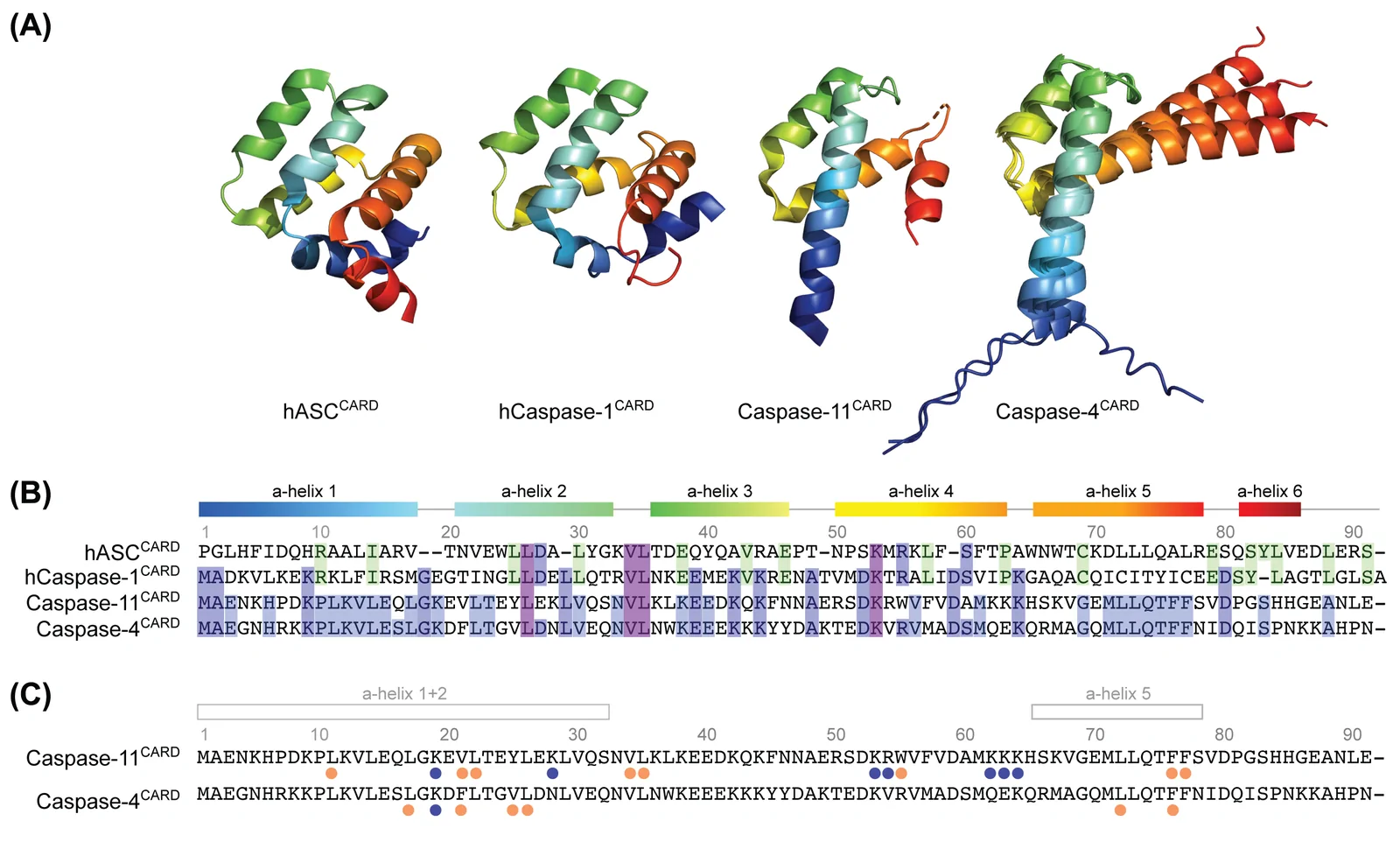
Alignment of inflammasome-related CARD domains (**A**) Ribbon representation of the three-dimensional structures of CARD domains from human ASC protein (PDB code: 7KEU), human caspase-1 (PDB: 5FNA), and mouse caspase-11 (PDB: 6KXG), and AlphaFold3 model of the CARD domain of caspase-4. (**B**) Sequence alignment of the hASC, hCaspase-1, Caspase-11, and Caspase-4 CARD domains. Alpha-helices are indicated and colors correspond to the helices shown in panel (A). Residues conserved across all CARD domains are in magenta; residues conserved in ASC and Caspase-1 CARDs only are shown in green; residues present in at least one LPS-binding CARD are shown in violet, and residues specific for LPS-recognizing CARDs only are shown in blue. (**C**) Residues experimentally shown to participate in LPS-driven activation of caspase-4/-11 are marked with orange (hydrophobic) or blue (charged) circles.

Structurally, the CARD belongs to the death-fold superfamily, a group of protein–protein interaction modules that also includes the pyrin, death effector, and death domains (Figure 1A) [30,31]. Classically, these domains mediate protein–protein interactions, necessary for complex assembly or signal transduction in pro-inflammatory or cell death pathways [32,33]. Normally death-fold domains, such as the CARDs of human caspase-1 or ASC, have a conserved compact structure, formed by six alpha helices (α1–α6) [30] (Figure 1A). In contrast, the crystal structure of MBP-tagged mouse caspase-11^CARD^ suggests that it adopts a distinct semi-outstretched conformation, composed of three core amphipathic α-helices (α3–5), while helices α1–2 are merged into a single longer helix facing outwards and helix α6 is not visible at all (Figure 1A) [34]. No experimental structural information for human caspase-4^CARD^ has been published so far, however, its AlphaFold3 model predicts a similar overall architecture, though with variable degrees of flexibility within N-terminal helix α1 and C-terminal helices α5–6, which have a tendency to kink and fold back, forming more compact bundle (Figure 1A). Moreover, the notion of a fully folded caspase-11^CARD^ has been challenged by two recent biophysical studies that employed circular dichroism (CD), nuclear magnetic resonance (NMR), and hydrogen-deuterium exchange mass spectrometry (HDX-MS) [35,36]. Intriguingly, the authors suggest that caspase-11^CARD^ is largely unstructured in solution, with only short segments transiently adopting helical secondary structure. Notably, these studies find that caspase-11^CARD^ undergoes a substantial structural transition upon incubation with LPS, approximately doubling its α-helical content. Despite this increase, the domain remains conformationally heterogeneous, leading the authors to propose that the LPS-bound caspase-11^CARD^ adopts a molten-globule-like state, i.e. a partially folded intermediate state with native-like secondary structure elements but a highly dynamic, fluid tertiary structure [36,37]. This could explain why no crystal or cryo-EM structures of the LPS/caspase-4(-11) complexes have yet been obtained. Therefore, the ability to crystallize caspase-11^CARD^ may be due to the stabilization effect of soluble N-terminal MBP tag [34].

The same studies also examined the stoichiometry of LPS/caspase-11^CARD^ complexes and reported heterogeneous assemblies containing between four and eight CARD domains and a varying number of LPS molecules. It is important to note that these experiments were performed in buffers containing detergents or chelators of divalent cations, which destabilize the tubular LPS micelles, allowing the dispersion of micelles into smaller LPS/caspase-4(-11) complexes [35,36]. In physiologically more relevant detergent-free conditions, caspase-4^CARD^ was binding to intact elongated tubular LPS micelles rather than facilitating their fragmentation. Strikingly, caspase-4^CARD^ binding occurred almost exclusively at LPS micelle ends, suggesting a preference for more complex regions with increased positive membrane curvature rather than for soluble LPS molecules, a conclusion supported by molecular dynamics simulations [22]. Such an association of caspase-4 with LPS membranes is consistent with infection studies showing recruitment of caspase-4 to the surface of cytosolic bacteria within infected cells [23–25]. It can also not be excluded that the interaction of the CARD with LPS changes LPS-containing membranes to create or promote positive curvature to facilitate activation of the caspase protease domain. How the interaction of caspase-4/11^CARD^ with LPS molecules at micelle ends or at positively curved membrane regions looks detail is unknown, but it is conceivable that it might also involve a molten-globule-like state of the CARD [22,35,36].

Together, these studies reveal fundamental differences between the CARDs of caspase-4 or caspase-11 and other members of the death-domain superfamily. Unlike classical death-fold domains, which are well-folded protein–protein interaction modules that drive the assembly of higher-order signaling platforms (e.g. canonical inflammasome, apoptosome, or PIDDosome complexes) [31–33,38–44], the caspase-4/11^CARD^ appears to have evolved into an LPS recognition module that adopts a partially unstructured conformation (Figure 1A). This unique feature allows the caspase to use LPS-containing membranes as non-proteinaceous oligomerization platforms or SMOCs (supramolecular organizing centers) [45–47]. In such a model, caspase-4/11 clustering on LPS-rich membranes or smaller LPS-containing vesicles would locally increase its concentration to levels that allow the dimerization of catalytic p20/p10 domains, an event that would otherwise be highly improbable given the low cytosolic abundance of caspase-4/11 (estimated at 10–60 nM) [44,48–51]. Consistent with this model, recent work has shown that CARD-mediated binding to LPS generates local p20/p10 concentrations within the range of the dimerization Kd, thereby promoting autoprocessing and formation of the active heterotetramer [36].

## Functional regions within the caspase-4/11^CARD^ that mediate LPS binding

Lipid A has been identified as the minimal structural component of LPS required to elicit non-canonical inflammasome activation [11,12]. Most commonly, lipid A carries six acyl chains, each about 14 carbons in length, two of which are secondary acyl chains attached to primary acyl chains [52–54]. Consistent with the presence of both hydrophobic region and negatively charged phosphate groups in lipid A, several corresponding positively charged and hydrophobic residues within the caspase-4/11^CARD^ domain have been identified to mediate LPS binding (Figure 1B,C) [12,22,35,55,56]. For instance, two recent independent HDX-MS analyses have shown that LPS binding results in altered deuteration of the very N-termini of caspase-4/11^CARD^ [22,35]. This region contains positively charged residues, among them K19, which is highly conserved and essential for caspase-11 binding to LPS *in vitro* and for pyroptosis induction in cells (Figure 1B) [12,24]. Another positively charged residue, K28, conserved in rodents, also maps to this region (Figure 1C) [56]. Underscoring the importance of positive charges in these positions, introducing the mutations D20K and E28K into the CARD of the cichlid fish *Amphilophus citrinellus*, which normally cannot bind LPS, was sufficient to restore LPS-binding capability [56]. The same N-terminal region contains several highly conserved hydrophobic residues that are shared among caspase-11, caspase-4, and caspase-5 homologs across different species (Figure 1C). Mutating these residues disrupts LPS binding by caspase-4/11^CARD^, abolishes oligomer formation, and prevents caspase-4 recruitment to cytosolic bacteria, indicating their essential functional roles [22,35]. A second hydrophobic region is also partially protected from deuteration in HDX-MS, although perturbing this site has a more modest effect on caspase-4/LPS interactions (Figure 1C) [22,35,56]. Intriguingly, despite NMR spectroscopy predicting the CARD to be largely unstructured, these two hydrophobic regions show the highest propensity to form secondary structure elements [36]. In the AlphaFold model, they correspond to helices α2 and α5, which may be a part of a putative hydrophobic pocket (Figure 1). Thus, regardless of whether the caspase-4/11^CARD^ is highly dynamic or adopts a more rigid conformation, the hydrophobic regions are the most likely elements to establish direct contacts with the hydrophobic acyl chain portion of LPS membrane. Overall, whether these regions engage sequentially, with the positively charged residues mediating the initial electrostatic contact with the negative charges in lipid A and thereby facilitating stronger hydrophobic interactions with the acyl chains, remains to be determined.

## Differential activation of caspase-4/-11 by different LPS variants

**Figure 2:**
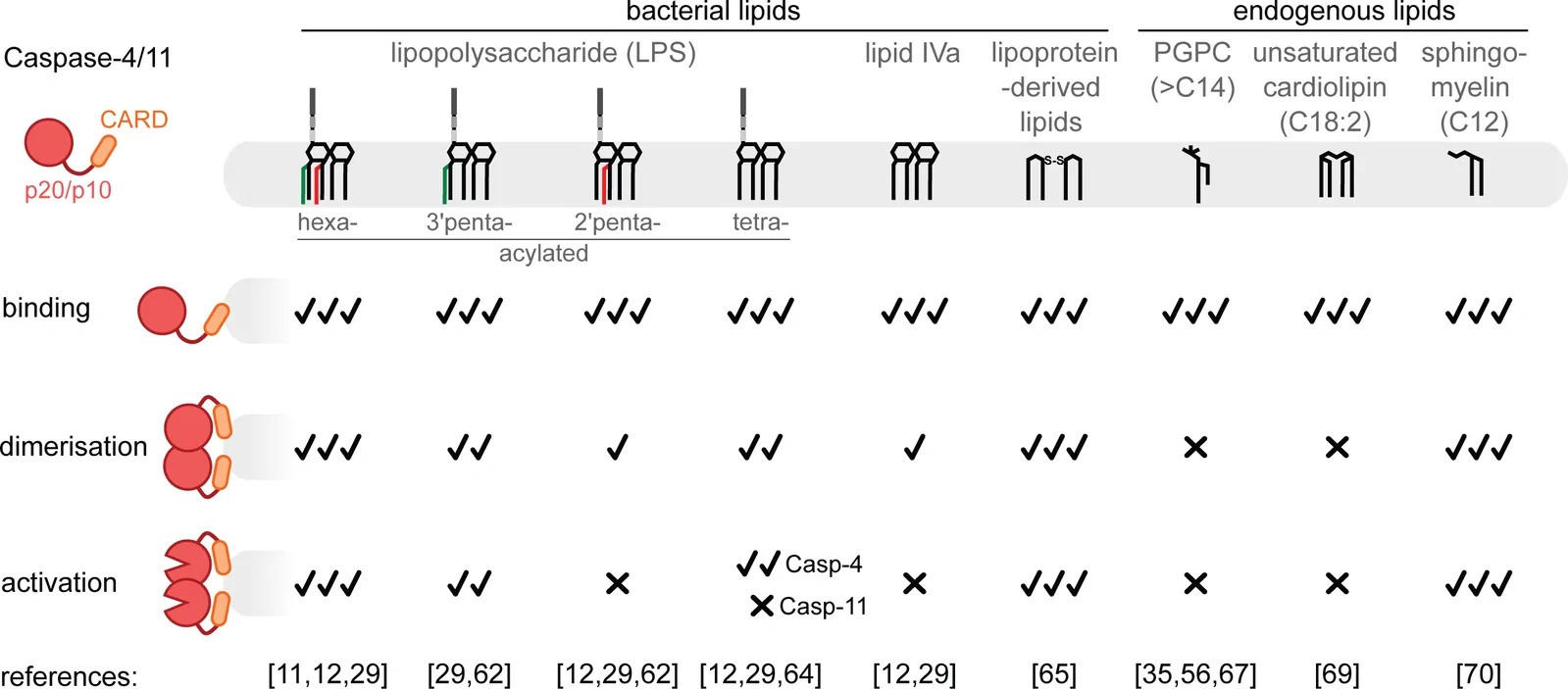
Ligand specificity of caspase-4/11 Summary of bacterial and endogenous lipid species that interact with caspase-4/11. Binding of caspase-4 to a membrane containing a given lipid leads to caspase dimerization and autoactivation. The efficiency of lipid binding, dimerization and activation induction is indicated by one to three ticks, reflecting how robustly each process is induced; a cross indicates that the given lipid fails to induce or inhibits the process.

Certain bacterial species actively modify their LPS layer to escape their recognition by TLR4 and caspase-4/-11 [2,57,58]. Lipid A is primarily acylated via hydroxyl groups at positions 2, 2′, 3, and 3′ of two monosaccharides, while the additional two secondary acyl chains are added to the acyl chains of the 2′ and 3′ positions [59]. One important mechanism for immune escape is the deacylation of LPS from this canonical hexa-acylated form to penta-acylated or even tetra-acylated variants [60–63]. Early studies using biotin-precipitations and pore-limiting gels, for example, showed that penta-acylated *Rhodobacter sphaeroides* LPS and the tetra-acylated LPS precursor lipid IVa bound the caspases-4/11, but failed to induce their oligomerization and consequently their autoactivation (Figure 2) [12]. More recent work examining the oligomerization state of the caspase-4/-11^CARD^ domains in response to different LPS species by size-exclusion chromatography, native mass spectrometry and mass photometry, confirmed that penta-acylated *R. sphaeroides* and *Porphyromonas gingivalis* LPS, as well as tetra-acylated lipid IVa, were capable of interacting with the caspase-4/11^CARD^ domain (Figure 2). The study also found that these ligands induced the formation of CARD/LPS complexes, but that these were smaller than complexes induced by hexa-acylated *Escherichia coli* or *Salmonella minnesota* LPS and did not trigger caspase-4/-11 activation *in vitro*. Nevertheless, these underacylated (<6 acyl chains) LPS species still induced conformational changes within the CARD domain, suggesting that they bind to the similar interface as the hexa-acylated LPS [35]. Together, these findings indicate that the number of LPS acyl chains is the key feature for non-canonical inflammasome activation (Figure 2). The position of the acyl chains might be another critical determinant, since analysis of caspase-11 activation by LPS derived from different *Yersinia pestis* lipid A mutants showed that penta-acylated LPS lacking the secondary acyl chain attached to the 2′ primary acyl chain (Δ*lpxP* bacteria) induced stronger activation than penta-acylated LPS lacking the secondary acyl chain attached to the 3′ primary acyl chain (Δ*msbB* bacteria) (Figure 2) [62]. In addition, differences in ligand specificity between caspase-4 and caspase-11 may exist, as tetra-acylated LPS from *Francisella novicida* was shown to activate caspase-4 in human macrophages but failed to induce caspase-11 activation in mouse bone marrow-derived macrophages, suggesting that caspase-4^CARD^ may recognize a broader range of LPS structures than its murine counterpart (Figure 2) [64].

## Lipids modulating the non-canonical inflammasome

Apart from recognizing LPS of Gram-negative bacteria, there is only limited evidence that caspase-4/-11 respond to other ligands or types of pathogens, such as Gram-positive bacteria or parasites. A recent study has, however, found that caspase-11 can be activated by four-acyl-chain-containing molecules (Figure 2), structurally resembling Pam3CSK4, derived from Gram-positive bacterial lipoproteins upon action of host cystathionine γ-lyase [65]. Mounting evidence, on the other hand, suggests that caspase-4/-11 can bind certain endogenous lipids *in vitro* that share structural features with LPS, that is, negatively charged headgroups and acyl chains longer than 14 carbons (C14+) (Figure 2) [35,56,66–70]. Importantly, such binding does not necessarily result in caspase-4/11 activation and could thus represent an endogenous suppressive mechanism. Oxidized phosphatidylcholines (OxPAPC), a mixture of oxidized self-lipids reported to bind CD14, TLR4, and MD-2, are the best-characterized endogenous ligands of caspase-4/11 [66–68]. Notably, PGPC (1-palmitoyl-2-glutaryl phosphatidylcholine), a component of OxPAPC, binds caspase-11 without inducing its aggregation or activation [56]. Moreover, this binding prevents LPS-induced aggregation of the caspase-11^CARD^ (Figure 2) [56]. Consistently, *in vivo* work showed that PGPC can suppress LPS lethality in mouse models [67]. A more detailed analysis by HDX-MS revealed that PGPC engages the same hydrophobic pocket/residues within the caspase-11^CARD^ as LPS (Figure 1C) [35]. It is thus likely that occupying this hydrophobic pocket antagonizes the recruitment of caspase-11 to LPS-containing membranes and thus its downstream activation. Another antagonist of caspase-4/-11 is mitochondrial di-unsaturated 18:2 cardiolipin (uCL), which has also been shown to compete with LPS for caspase-4/11^CARD^ binding and to suppress LPS-induced caspase-4/11 activation (Figure 2) [69]. Both PGPC and uCL display features that are crucial for interaction with caspase-4/11^CARD^, i.e. negatively charged headgroups and C14+ acyl chains, thus supporting the recently proposed model that the caspase-4/11^CARD^ acts as a bipartite lipid-binding module [56]. The fact that they do not activate but rather suppress caspase-4/11 activation might be due to their higher solubility compared with LPS, which may allow binding without inducing clustering of the catalytic domains *in vitro*. Interestingly, another endogenous lipid, sphingomyelin d18:1/14:0 (SM C12), which is produced during meta-inflammation and can activate TLR4, has recently been shown to activate the non-canonical inflammasome in mouse macrophages (Figure 2) [70]. Molecular docking showed that several molecules of SM C12, which has two acyl chains, are needed to occupy the LPS-binding pocket in TLR4/MD-2 [70]. Yet how SM C12 interacts with caspase-4/11 and how it induces their activation remains to be determined.

Overall, these findings imply that caspase-4/11 are not specific sensors of LPS but have a much broader ligand-binding spectrum than previously anticipated. In future, the list of potential modulators of caspase-4/11 activity might be further expanded since *in vitro* studies have shown that caspase-11^CARD^ can additionally bind phosphatidyl inositol phosphates (PIPs) [56]. The key question that needs to be answered is whether these ligands act as suppressors or activators of inflammatory caspases—i.e. whether they keep the caspases monomeric or induce their clustering and autoactivation. It is conceivable that local high concentrations of these lipids, coupled with positive membrane curvature, could be deciding factors that shift the balance from suppression to activation.

## Enhancement of LPS/caspase-4 (-11) interaction by guanylate-binding proteins

**Figure 3:**
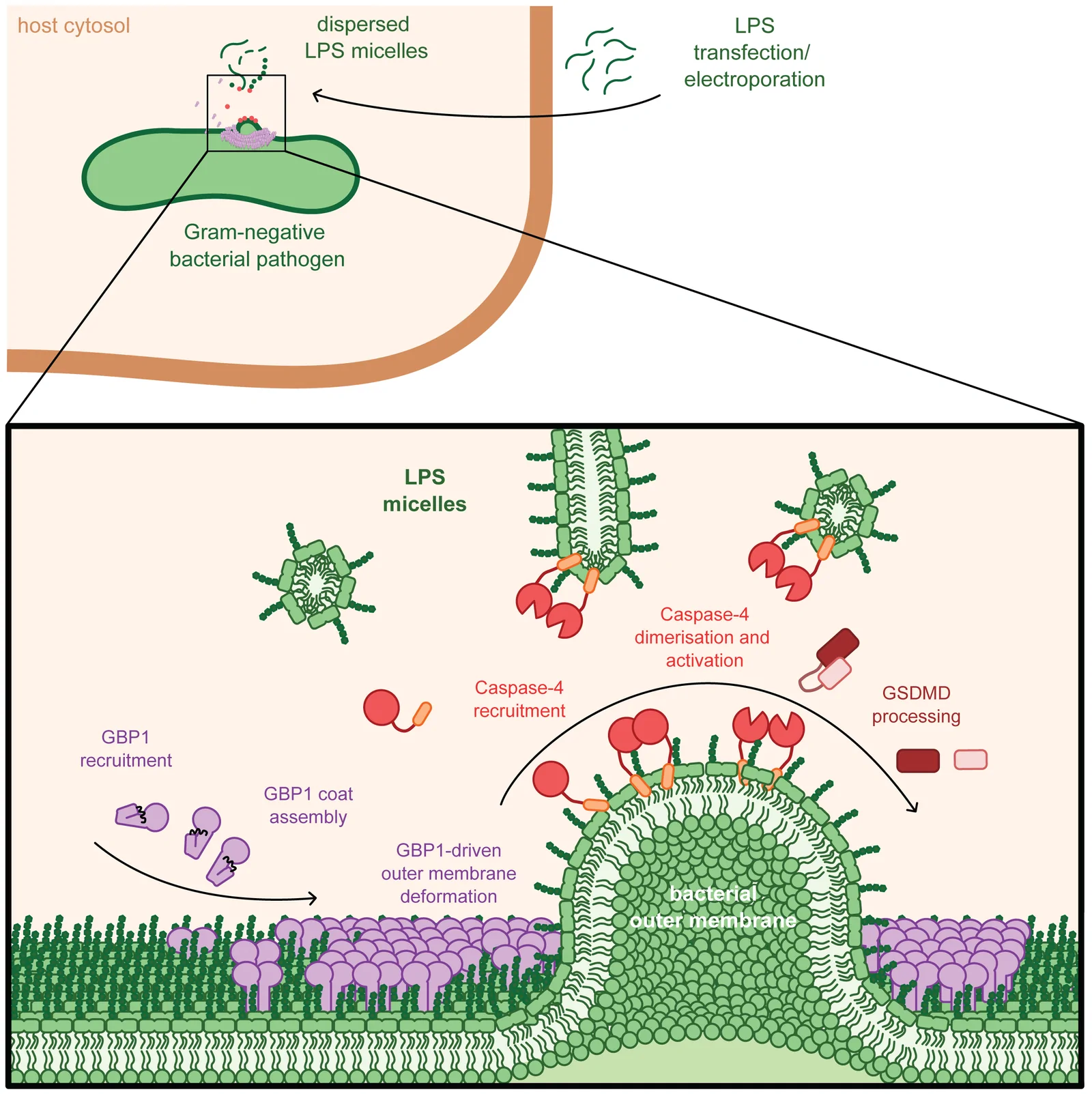
Model for non-canonical inflammasome assembly in cells In humans, cytosolic Gram-negative pathogens, such as *Salmonella* or *Shigella* species, are recognized by GBP1, which directly binds LPS in their outer membrane, leading to the formation of an oligomeric GBP1 coat that also includes GBP2–4 (not shown). The mechanoenzyme activity of GBPs deforms the outer membrane, creating regions of positive curvature that expose the hydrophobic portions of LPS. This exposure enables efficient recruitment of caspase-4 monomers to the bacterial surface and promotes local clustering of the caspase—a prerequisite for caspase-4 dimerization, autoactivation, and processing of its substrate GSDMD. Electroporation or transfection of purified LPS delivers LPS micelles in a dispersed form that is more readily accessible to caspase-4, partially bypassing the requirement for GBPs. LPS membrane, GBP1, caspase-4, and GSDMD are shown in green, magenta, red-orange, and brown, respectively.

Several studies have shown that priming with type I or type II IFN enhances caspase-4/11 activation independently of its effects on caspase expression, thus implying that interferon-stimulated genes might positively regulate non-canonical inflammasome activation. Consistent with this, several groups reported that during bacterial infection, caspase-4 or caspase-11 activation depends on guanylate-binding proteins (GBPs) [71–74], a family of IFN-induced large GTPases that, based on their structure, belong to the dynamin superfamily and comprise seven members in humans and eleven in mice [23,24,75–81]. Moreover, GBP-deficiency was shown to protect against LPS-induced lethality in mice, even though the effect was less pronounced than that observed in caspase-11 or GSDMD-deficient animals [11,75,82]. At the molecular level, GBPs assemble into coat-like structures around intracellular bacteria [23–25,77,83–86]. Studies dissecting the human GBP family have shown that GBP1 targets the bacterial outer membrane through direct binding to LPS, which subsequently recruits GBP2, -3, and -4 and ultimately promotes the recruitment and activation of caspase-4 on GBP-coated bacteria [23–25]. Recent structural and biochemical studies have further expanded our understanding of caspase-4/11^CARD^-mediated LPS recognition, and the role of GBPs herein [22,84–86]. GBP1 has been shown to function as a mechanoenzyme capable of deforming LPS-containing membranes, including the outer membrane of Gram-negative bacteria, thereby generating membrane regions with distinct positive and negative curvatures (Figure 3) [22,87]. Given the preferential binding of caspase-4^CARD^ to positively curved LPS membranes [22], this observation may provide a mechanistic explanation for the requirement of GBP1 in facilitating caspase-4 activation during Gram-negative bacterial infections (Figure 3). To fully confirm this model, it will be important to analyze the spatial organization between GBPs and caspase-4 coats on the outer bacterial membrane or on transfected LPS micelles *in situ*, using high-resolution methods such as Cryo-ET.

## Evolution and expression of human caspase-4/-5 and mouse caspase-11

**Figure 4:**
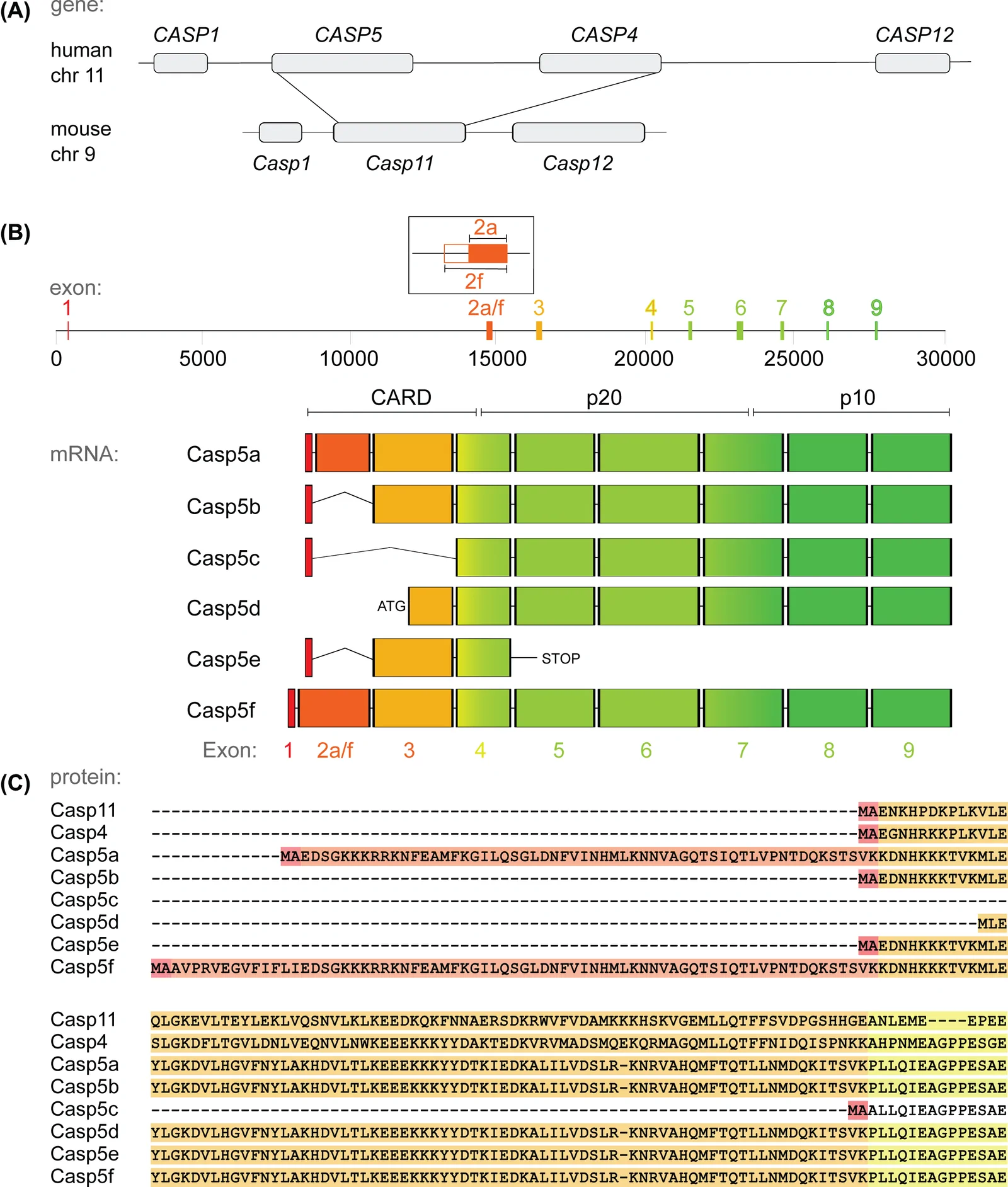
Evolution of *CASP4/5* gene locus Organization of (**A**) gene loci coding for inflammatory caspases in human and mice, (**B**) caspase-5 exons, with regions coding for CARD, p20, and p10 subunits being highlighted, and (**C**) alignment of amino acid sequences of CARD domains (corresponding to exons 1–3) of caspase-11, caspase-4, and caspase-5a-5f.

Caspase-4, -5, and -11 belong to the subfamily of inflammatory caspases, which are encoded in a locus on human chromosome 11 and mouse chromosome 9 together with caspase-1, the executioner of the canonical inflammasome, as well as caspase-12 (Figure 4A) [88–91]. Evolutionary analyses indicate that caspase-4 and caspase-5 are orthologs of murine caspase-11, arising from a primate-specific gene duplication event [92,93]. Several caspase-5 isoforms have been identified at RNA level, designated caspase-5a to caspase-5f (Figure 4B) [94,95], with isoforms a–c also detected at the protein level in human monocyte-derived macrophages and human intestinal epithelial cells (IECs) [96]. Interestingly, these isoforms mainly differ in the length and composition of their CARD (Figure 4B,C). Caspase-5b features a CARD that is most similar to the one of caspase-4 (Figures 1A, 4C), whereas caspase-5a and caspase-5f possess an extended CARD resulting from the inclusion of an additional exon (Figure 4C). In contrast, caspase-5c lacks the CARD entirely, while caspase-5d contains a slightly truncated version (Figure 4C) [94]. Comparative analysis of inflammatory caspase gene clusters across species also suggested that while caspase-11 and -4 are conserved, caspase-5 shows indications of positive selection, particularly within the extended N-terminal CARD domain found in caspase-5a/-5f as well as its catalytic p20 domain [93]. Two of the positively selected residues within the caspase-5 p20 domain, V217 and R221, are located close to the binding site of OspC2, a recently identified effector of *Shigella flexneri* that specifically targets and inactivates caspase-5, but not caspase-4 [97]. This observation suggests that adaptive changes within the caspase-5 p20 domain may have arisen as part of a host-pathogen evolutionary arms race. Whether a similar selective pressure might shape the positively selected residues identified within the caspase-5 CARD domain remains unknown. Notably, several of these residues map to a region that may be targeted by NleL, a HECT-like E3 ubiquitin ligase produced by *Citrobacter rodentium* and enterohaemorrhagic *Escherichia coli*. NleL promotes the proteasomal degradation of caspase-11, -4, and -5, but interestingly, not of caspase-1. Replacing residues 6–20 of its CARD domain with the corresponding sequence from caspase-4 renders caspase-1 susceptible to degradation, indicating that the very N-terminal region is a key determinant of NleL sensitivity [98].

The observation that caspase-4 and caspase-5 have been evolving in parallel in primate genomes suggests they may be required during different pathophysiological states rather than being functionally redundant [93]. This is also supported by distinct RNA expression patterns available in Human Protein Atlas: *CASP4* transcripts are ubiquitously expressed across tissues, whereas *CASP5* expression is largely restricted to the intestinal tract and appendix (Table 1). In contrast, *Casp11* displays a broad expression profile that closely resembles that of *CASP4* [99,100]. The expression of these inflammatory caspases is further enhanced during infection. In particular, IFN signaling, and to a lesser extent LPS-induced TLR4 signaling, are critical for the up-regulation of *Casp11* transcripts in mice and *CASP5* expression in human cells, whereas *CASP4* appears to be largely constitutively expressed (Table 1) [9,10,101–106].

**Table 1:** Features of pro-inflammatory caspases-4, -5, and -11

Feature:	caspase-4	caspase-5	caspase-11	Ref.
**Tissue distribution**	*broad*	*restricted*	*restricted*	Human Protein Atlas
**Expression level**	*constitutive*	*low, inducible (IFN/ LPS)*	*very low, inducible (IFN)*	[10,96,103,106,111]
**Regulation by ISGs**	*GBP1-4*	*n.d.*	*Gbp^chr3^, IRGM; IRGB10*	[23,24,73–75,77,112]
**Binds LPS *in vitro***	*yes*	*yes (casp-5a/f)*	*yes*	[12,21,22]
**Coats bacteria in cells**	*yes*	*n.d.*	*n.d.*	[23–25]
**LPS-induced pyroptosis**	*yes*	*n.d.*	*yes*	[16,82]
**Cleaves Gasdermin D**	*yes*	*n.d.*	*yes*	[13,16,82]
**Cleaves pro-IL-1β**	*yes*	*n.d.*	*no*	[9,113–115]
**Cleaves pro-IL18**	*yes*	*n.d.*	*no*	[17,18,23,24,106]

Regarding caspase function, it is now well established that caspase-4 and -11 cleave GSDMD to induce pyroptosis, and that caspase-4, but not caspase-11, can process IL-1 cytokines (Table 1). Yet, comparably little is known about the biological function of caspase-5 and its isoforms [95,107–110]. A recent study reported that cytosolic LPS predominantly activates caspase-4 in human monocyte-derived macrophages and IECs, despite detectable expression of caspase-5 [96]. This observation raises questions about the physiological importance of caspase-5 *in vivo* and whether LPS is indeed its primary ligand. Interestingly, the same study reported that the CARD-less isoform caspase-5c promotes Wnt signaling in IECs by interacting with the protein Dishevelled and cleaving the central scaffold protein APC (adenomatous polyposis coli) [96]. These findings raise the possibility that caspase-4 and caspase-5 do not primarily differ in their capacity to sense LPS, but rather in their substrate repertoires, thus controlling distinct signaling outputs.

## Concluding Thoughts

The non-canonical inflammasome pathway, mediated by caspase-4 in humans and caspase-11 in mice, is now firmly established as a direct sensor of cytosolic LPS. Nevertheless, significant questions remain regarding both the structural and functional aspects of this pathway. For example, additional insight into the structure of full-length caspase-4, before and after autoprocessing, in complex with LPS-containing membranes is still lacking. Although the highly dynamic nature of the CARD may preclude its high-resolution structural characterization, understanding how the full-length protein assembles on membranes would substantially advance our knowledge of caspase activation mechanisms.

Another major unresolved question concerns the architecture of caspase-4 complexes assembled on the surface of cytosolic bacteria. Specifically, it remains unclear what the spatial organization of GBPs, the caspase, and the bacterial membrane is, and which features of the native bacterial outer membrane (such as membrane composition, local curvature, and membrane perturbations) actually promote caspase recruitment and activation. Furthermore, it is unknown whether activated caspase complexes remain associated with bacterial surfaces or whether they are released into the cytosol following activation.

The physiological function of caspase-5 also remains poorly understood. Although its CARD domain binds LPS *in vitro*, compelling evidence for a role in host defense is lacking, raising questions about its biological relevance and function. One possibility is that caspase-5 recognizes distinct pathogens or specific LPS structures that are not efficiently sensed by caspase-4. Alternatively, caspase-5 may primarily mediate functions beyond canonical GSDMD cleavage and IL-18 release, potentially contributing to other signaling pathways, as recently suggested. In this regard it will be important to further characterize the substrate repertoire of inflammatory caspases.

Additional work is also needed to identify and characterize other ligands capable of engaging the CARD domains of caspase-11, caspase-4, and caspase-5. In this context, it will be particularly interesting to determine whether all endogenous lipids that bind the CARD act as competitive inhibitors of LPS sensing and thereby suppress caspase activation, or whether some endogenous lipids can themselves function as bona fide activators. Such a scenario would substantially expand the current view of these inflammatory caspases. Rather than serving primarily as sensors of microbial invasion, caspase-4/-5/-11 may have evolved more broadly to monitor disturbances in lipid homeostasis or membrane integrity.

Finally, it remains to be determined whether new insights into the mechanism of LPS-CARD interaction can be translated into novel therapeutic strategies to suppress caspase-4 activation during Gram-negative bacterial sepsis. In mouse models, the pathology of Gram-negative sepsis has been linked to excessive activation of the non-canonical inflammasome. Therapeutic approaches aimed at inhibiting GBP function or blocking access of LPS to the ligand-binding region within the CARD could complement existing strategies that target the catalytic activity of inflammatory caspases.

## Perspectives

- The binding of the CARD domain to LPS is well documented; however, the underlying structural basis of this interaction remains uncharacterized. Elucidating how LPS is recognized and determining the conformational changes it induces in caspase-11/4 will be critical for advancing our understanding of non-canonical inflammasome activation.
- In humans, two inflammatory caspases, caspase-4 and caspase-5, have been shown to interact with LPS *in vitro*. However, the physiological function of caspase-5 remains largely unknown. Is caspase-5 a homolog of caspase-4 with a more restricted expression profile, or does it possess unique properties, such as distinct ligands, substrates, or signaling functions? Furthermore, which of the reported caspase-5 isoforms are biologically relevant, and under what physiological or pathological contexts do they operate?
- The repertoire of lipid ligands reported to interact with the CARD domain has recently expanded, particularly with the identification of endogenous lipid species. However, the functional significance of these interactions remains poorly understood. Do these endogenous ligands primarily act as negative regulators that suppress caspase-4/11 activation, or might some function as endogenous activators, serving as indicators of disturbed lipid homeostasis, membrane damage, or metabolic stress?

## Open Access

Open access for this article is provided via our Subscribe to Open programme, made possible through the support of our subscribing institutions. Learn more here: https://portlandpress.com/pages/open_access_options_and_prices#conditional.

## Abbreviations

CARD: caspase recruitment domain
CD: circular dichroism
GBPs: guanylate-binding proteins
GSDMD: gasdermin-D
HDX-MS: hydrogen-deuterium exchange mass spectrometry
IEC: intestinal epithelial cells
IFN: interferons
LAL: Limulus amebocyte lysate
LPS: lipopolysaccharide
NMR: nuclear magnetic resonance
OxPAPC: oxidized phosphatidylcholines
PAMPs: pathogen-associated molecular patterns
PGPC: 1-palmitoyl-2-glutaryl phosphatidylcholine
PRRs: pattern recognition receptors
TLR4: Toll-like receptor 4
